# Characteristics and outcomes of patients with HER2-negative metastatic breast cancers with low expression of estrogen and progesterone receptors in the multicenter ESME cohort

**DOI:** 10.1016/j.breast.2026.104815

**Published:** 2026-05-18

**Authors:** Marie Alexandre, Eva Teruel, Florence Dalenc, Thibaut de la Motte Rouge, Etienne Brain, Vincent Massard, Monica Arnedos, Audrey Mailliez, Jean-Marc Ferrero, Thomas Bachelot, Isabelle Desmoulins, Marie-Ange Mouret-Reynier, Christelle Levy, Antony Gonçalves, Anca Berghian, Thierry Petit, Jean-Sébastien Frenel, Anne-Laure Martin, Thomas Grinda, William Jacot

**Affiliations:** a Department of Medical Oncology, Regional Cancer Institute, Montpellier, France; b Biostatistics Unit, Regional Cancer Institute, Montpellier, France; c Department of Medical Oncology, Oncopole Claudius Regaud IUCT-O, Toulouse, France; d Department of Medical Oncology, Centre Eugène Marquis, Rennes, France; e Department of Medical Oncology, Institut Curie, Saint-Cloud, France; f Department of Medical Oncology, Institut de Cancérologie de Lorraine, Vandœuvre-lès-Nancy, France; g Department of Medical Oncology, Institut Bergonié, Bordeaux, France; h Department of Medical Oncology, Centre Oscar Lambret, Lille, France; i Department of Medical Oncology, Centre Antoine Lacassagne Université Côte d’Azur, Nice, France; j Department of Medical Oncology, Centre Léon Bérard, Lyon, France; k Department of Medical Oncology, Centre Georges-François Leclerc, Dijon, France; l Department of Medical Oncology, Centre Jean Perrin, Clermont-Ferrand, France; m Department of Medical Oncology, Centre François Baclesse, Caen, France; n Department of Medical Oncology, Institut Paoli-Calmette, Marseille, France; o Department of Medical Oncology, Centre Henri Becquerel, Rouen, France; p Department of Medical Oncology, Centre Paul Strauss ICANS, Strasbourg, France; q Department of Medical Oncology, Institut de Cancérologie de l’Ouest, Saint-Herblain, France; r Health Data and Partnership department, Unicancer, Paris, France; s Breast Cancer Unit, Gustave Roussy, Villejuif, France

**Keywords:** Metastatic breast cancer, Hormone receptor low, Real-world data, Overall survival

## Abstract

**Background:**

Breast cancers expressing low (1-9%) immunohistochemistry levels of estrogen and progesterone hormone receptors (HR), remain an uncertain category, being considered either as triple negative (TN) breast cancers or HR-positive (HR+) tumors across guidelines or approvals.

**Methods:**

ESME is a National real-world cohort of all consecutive patients who initiated a first-line treatment for metastatic breast cancer (MBC) from year 2008 onwards in one of the 18 French Comprehensive Cancer Centers. We analyzed baseline data and outcomes from all patients with HER2-negative MBC and known HR expression levels. Our primary objective was to evaluate overall survival (OS) in HR-low MBC patients compared with those with TN and HR + disease.

**Results:**

Out of 30,459 patients in the ESME database who initiated an MBC treatment between 01/2008 and 01/2021, 19,109 were eligible for this analysis: 16768, 2113, 228 respectively with HR+, TN, and HR-low MBC. Median follow-up was 58.0 months (56.6-59.0).

Median OS were 44.6 (43.8-45.5), 19.1 (15.5-22.4) and 15.7 (15.0-16.8) months in the HR+, TN, and HR-low groups, respectively. The multivariable analysis identified no difference in OS between HR-low and TN groups (Hazard Ratio 0.93, 95%CI 0.77-1.11). Median PFS under first line chemotherapy were 10.2 (9.9-10.6), 5.3 (5.1-5.6) and 5.1 (4.1-6.2) months for HR+, TN and HR-low groups, respectively. In the multivariable analysis, the HR-low group had a statistically poorer PFS compared to the TN group (Hazard Ratio 1.24, 95% CI 1.04-1.49).

**Conclusions:**

In this large cohort, patients with HR-low MBC have the same dismal overall survival as those with TN MBC.

## Highlights

• Median OS were 44.6 (43.8-45.5), 19.1 (15.5-22.4) and 15.7 (15.0-16.8) months in the HR+, triple-negative, and HR-low groups.
• The prognosis of patients with HR-low tumors was similar to the prognosis of patients with triple-negative tumors.
• HR-low MBC patients should probably be considered as triple-negative in term of risk stratification and therapeutic options.

## 1 Introduction

In breast cancer, the expression levels of hormone receptors (HR, whether Estrogen Receptor [ER] or Progesterone Receptor [PR]) are evaluated by immunohistochemistry (IHC), with results reported as the proportion (%) and intensity of positively stained tumor cells (based on the Allred scoring) [1]. In clinical practice, HR variable is then qualified as HR positive (HR+) or negative according to a threshold, whether 1% in the US guidelines or 10% in European guidelines. However, the exact impact of the variations in the level of expression of ER and PR and their impact on clinical outcome is poorly documented in the metastatic setting.

The position of clinical practice guidelines for breast cancers expressing low (1-9 %, HR-low group) levels of HR is controversial. Currently, several guidelines define an ER-low group, with various implications. Indeed, according to ASCO guidelines, there is limited data on endocrine therapy (ET) efficacy for these patients but it suggests a possible benefit, justifying its prescription in the adjuvant setting [2]. NCCN guidelines note that there are more limited data about this population, which is heterogeneous and with clinical and biologic behavior often more similar to TN. They do not present global recommendations for this group, but propose a decision on a case-by-case basis [3]. In the metastatic setting, ESMO guidelines find limited evidence that these cancers may be less sensitive to ET but may benefit from combined ET and CDK4/6 inhibitors [4]. ABC5 guidelines consider that metastatic patients should not be treated with ET alone and could be included in triple negative (TN) breast cancer clinical trials [5].

Several studies questioned the place of this group, mainly in the situation of early breast cancer (EBC). In the seminal publication by Harvey and colleagues, the threshold of positivity of ER was set at 1% weakly staining tumor cells, with a statistically significant change in DFS in patients receiving ET [1]. Other studies are discordant regarding ET efficacy in the adjuvant setting [6,7]. Recent studies showed a profile closer to TN breast cancer than to HR ≥ 10% breast cancer in terms of clinical outcomes such as pCR rate after neoadjuvant chemotherapy or DFS [[8], [9], [10], [11], [12]]. Gene expression profiles in this HR-low group seemed to be more often related to TN group profiles than to cancers with HR ≥ 10% [13,14], even if the distribution seemed sometimes more heterogeneous than for TN [15]. In the neoadjuvant setting, immunotherapy seemed more efficient in this group than in HR ≥ 10% group [16].

The clinical issues regarding this group are both prognostic, with the need of a better definition of their clinical outcome and predictive as, theoretically, these tumors could be classified as candidates for ET depending on the guidelines used. In the same way, there is the question of access for these patients to treatments currently used in metastatic TN cancer but not in HR + cancer, such as immunotherapy.

In light of the limited data available in the metastatic setting, we aimed to investigate the prognosis and characteristics of HR-low group compared to TN and HR ≥ 10% groups, in a large French database of metastatic breast cancer (MBC) patients.

## 2 Methods

### 2.1 Design and population

The ESME MBC database is a French real-world database of all consecutive patients who initiated a first-line treatment for MBC from 2008 onwards at one of the 18 French Comprehensive Cancer Centers. The ESME MBC database (NCT03275311) was authorized by the French data protection authority (initial authorization no. DE-2013-117 and subsequent amendment in accordance with GDPR) and is managed by Unicancer. All patients have approved the use of their data.

Male or female patients, aged ≥18 years, with histologically proven metastatic breast cancer, HER2 negative, defined as non-overexpressed or non-amplified (0 to 2+, ISH negative) and known ER and PR receptors expression levels (percentage and intensity), initiating their MBC treatment between 01/2008 and 01/2021, were selected from the ESME MBC database. HR levels were defined from the latest available biopsy.

The patients' tumors were classified into 3 groups defined as follows: HR + group if ER and/or PR's expression were ≥10%, TN group if ER and PR's expression were <1%, and HR-low group if ER and PR's expression were both between 0 and 9% but not both <1%.

Data were extracted from the database about age at metastasis diagnosis, histological type, histological grade, HR and HER2 status according to IHC on adjuvant (if applicable) and metastatic setting, location of metastases, number of metastasis sites, germline BRCA mutations, metastasis-free interval, year of diagnosis, level of center activity and first line systemic therapy at the metastatic stage. In case of metachronous cancer, we looked at adjuvant treatment received for EBC. Location of metastases was defined as follows: non visceral metastasis (bones, nodes, pleura and/or skin, without brain or visceral metastasis), non-brain visceral metastasis (with or without non visceral metastasis) and brain metastasis (brain or meninge, with or without non visceral or visceral metastasis).

We planned an exploratory analysis on the ER-low group defined as HER2 non-overexpressed or non-amplified, ER expression between 1% and 9% BC and PR 0, compared with TN group and ER ≥ 10% group (supplementary data).

### 2.2 Objectives

Primary objective was to evaluate and compare overall survival (OS) in the 3 groups (HR+, TN and HR-low MBC patients).

Secondary objectives were to evaluate and compare progression-free survival (PFS) under first line chemotherapy between groups and to describe the clinical characteristics and the distribution of genetic abnormalities identified at the time of MBC diagnosis.

### 2.3 Endpoints

OS was defined as the time between the date of metastatic diagnosis and the date of death, whatever the cause.

PFS under first line treatment was analyzed in the subpopulation of patients who received chemotherapy as first treatment. PFS was defined as the time from the start of first chemotherapy to the date of death or first progression whichever occurred first. Progression consists in the diagnosis of either a local or loco-regional relapse, a new metastatic site or the progression of an existing metastasis occurring at least one month after the start of the first line, as reported in the database. If the records in the database indicate no explicit diagnosis of progression but a treatment termination due to progression, the patient was considered to have had a disease progression at the time of treatment withdrawal.

Patient without any event reported following first line treatment start date were censored at the date of the last medical information available.

### 2.4 Statistical analyses

Median of follow-up with its 95% confidence interval (95%CI) is described according to the reverse Kaplan-Meier method.

The Kaplan-Meier method was used to analyze survival data and to estimate rates and median survival times. The associated survival curves are presented. Survival distributions were compared by the Logrank test.

To assess the adjusted effect of HR status on OS and PFS, a full multivariable model including the variables below was fitted: age, histological grade, ER status at metastatic diagnosis, PR status at metastatic diagnosis, histology at initial diagnosis, HER2 status at metastatic diagnosis, Metastatic-Free interval, metastases types, number of metastatic sites, period of care. A backward selection strategy was then applied, checking for cofounding effect at each step as proposed by Hosmer and Lemeshow. HR status was forced in the model selection. The Hazard Ratios associated with each factor from the final model, with their 95% confidence interval are presented.

For the description of characteristics according to each HR group, categorical variables were described by the number of observations (n) and the percentages were calculated in relation to the total population excluding missing data.

## 3 Results

### 3.1 Patients and diseases characteristics

Out of 30,459 patients in the ESME database initiating their MBC treatment between 01/2008 and 01/2021, 19,109 patients (16,768 HR+; 2113 TN; 228 HR-low) were eligible for this analysis. Others were excluded due to HER2 status (n = 6905), undetermined HR status (n = 80) or unexploitable ER percentage (n = 4248) (Fig. S1 in supplementary data). Frequency of HR-low MBC represented 1.2% of all patients. Median follow-up was 58.0 months (95%CI [56.6; 59.0]). Patient population of the exploratory analysis of ER-low group is described in Fig. S2 in supplementary data.

Patient and tumor characteristics are described in Table 1. In HR-low group, 32.5% (n = 74) of patient were under 50 years old, a proportion similar to TN group (31.3%, n = 662), while patients in the HR + group were older (20.2% under 50 years, n = 3388). Brain metastasis occurred more frequently in HR-low group (14.0%, n = 32) and TN group (11.9%, n = 252) than in HR + group (3.8%, n = 636) while bone-only disease was less frequent in HR-low group (11.8%, n = 27) and TN group (10.3%, n = 217) than in HR + group (32.9%, n = 5524). Histology of HR-low breast cancer was mostly ductal (81.1%, n = 185), with a distribution more similar to TN (84.4%) than to HR + breast cancer (72.3%). For other histo-pathological characteristics, such as grade or level of HER2 expression in IHC, distribution in HR-low group was more heterogeneous than for TN group, with less grade 3 (56.6% vs 63.8%) and more HER2-low (38.6% vs 27.2%) tumors. The proportion of HR-low MBC was similar over time, averaging 1.2%.

**Table 1:** Patient and tumor characteristics.

	HR group					
	TN		HR low		HR +	
	N = 2113		N = 228		N = 16,768	
**Age at metastatic diagnosis**						
<50	662	(31.3%)	74	(32.5%)	3388	(20.2%)
[50 - 70]	1035	(49.0%)	111	(48.7%)	8628	(51.5%)
>70	416	(19.7%)	43	(18.9%)	4752	(28.3%)
**HER2 status (IHC score)**						
0	1535	(72.7%)	102	(61.4%)	9770	(58.7%)
1+	366	(17.3%)	35	(21.1%)	4151	(24.9%)
2+ (ISH negative)	209	(9.9%)	29	(17.5%)	2719	(16.3%)
Missing	3		62		128	
**Histology at initial diagnosis**						
Ductal	1776	(84.4%)	185	(81.1%)	11965	(72.3%)
Lobular	97	(4.6%)	19	(8.3%)	2755	(16.6%)
Both	9	(0.4%)	4	(1.8%)	167	(1.0%)
Other	223	(10.6%)	20	(8.8%)	1666	(10.1%)
Missing	8		0		215	
**Histological Grade III at primary tumor diagnosis**						
No	739	(36.2%)	96	(43.4%)	11967	(76.3%)
Yes	1300	(63.8%)	125	(56.6%)	3724	(23.7%)
Missing	74		221		1077	
**Type of metastases**						
Non visceral metastases	875	(41.4%)	105	(46.1%)	8802	(52.5%)
Including bone-only metastases	217	(10.3%)	27	(11.8%)	5524	(32.9%)
Brain metastases	252	(11.9%)	32	(14.0%)	636	(3.8%)
Including brain-only metastases	100	(4.7%)	12	(5.3%)	221	(1.3%)
Non brain visceral metastases	986	(46.7%)	91	(39.9%)	7330	(43.7%)
**Number of metastatic site at metastatic diagnosis**						
<2	1120	(53.0%)	138	(60.5%)	9496	(56.6%)
≥2	993	(47.0%)	90	(39.5%)	7272	(43.4%)
**Disease-free interval (months)**						
≤6 (De novo)	643	(30.4%)	72	(31.6%)	5642	(33.6%)
]6; 24]	679	(32.1%)	56	(24.6%)	1211	(7.2%)
]24; 72]	479	(22.7%)	55	(24.1%)	3914	(23.3%)
>72	312	(14.8%)	45	(19.7%)	6001	(35.8%)
**Germline *BRCA1* mutation at metastatic diagnosis**						
Mutated	96	(4.5%)	6	(2.6%)	70	(0.4%)
Not mutated	336	(15.9%)	40	(17.5%)	1151	(6.9%)
Not tested	1681	(79.6%)	182	(79.8%)	15547	(92.7%)
**Germline *BRCA2* mutation at metastatic diagnosis**						
Mutated	19	(0.9%)	3	(1.3%)	179	(1.1%)
Not mutated	374	(17.7%)	40	(17.5%)	1037	(6.2%)
Not tested	1720	(81.4%)	185	(81.1%)	15552	(92.7%)
Year of metastatic diagnosis, n (%)						
[2007-2009]	286	(13.5%)	24	(10.5%)	2274	(13.6%)
[2010-2012]	423	(20.0%)	45	(19.7%)	3983	(23.8%)
[2013-2015]	447	(21.2%)	63	(27.6%)	4455	(26.6%)
[2016-2018]	605	(28.6%)	59	(25.9%)	4190	(25.5%)
[2019-2021]	352	(16.7%)	37	(16.2%)	1866	(11.1%)

Six thousand three hundred and fifty-seven patients (33.3%) had synchronous metastases (diagnosis of MBC within 6 months of the primary tumor).

Among the patients with metachronous metastases (66.7%), 1211 HR+ (7.2%), 679 TN (32.1%) and 56 HR-low (24.6%) patients had an early relapse (between 6 and 24 months), whereas 6001 HR+ (35.8%), 312 TN (14.8%) and 45 HR-low (19.7%) patients had a late relapse (>72 months). Among the 156 HR-low patients who had metachronous disease, 129 (82.7%) had received chemotherapy and 57 (36.5%) ET as part of adjuvant treatment (Supplementary Table S1).

In the ESME database, HR levels are defined from the latest available biopsy. As consequence, the HR status may have been defined by the last metastasis biopsy or by the primary tumor status if there was no biopsy at the metastatic stage. Of the 228 HR-low patients, the phenotype was based on the metastasis in 109 patients (47.8%) and on the primary tumor in 119 patients. Among the 109 patients whose phenotype was based on metastasis, a phenotype based on the primary tumor was available for 84 patients. Of these 84 patients, 18 (21.4%) were already HR-low in the primary tumor, 50 (59.5%) were HR+, and 16 (19%) were TN (Supplementary Table S2). Among the 50 patients whose tumors switch from HR + to HR-low, 43 received ET as part of adjuvant treatment.

Known germline *BRCA1* mutations (at MBC diagnosis) were present in 0.4% of the HR + cases, 4.5% of the TN cases and 2.6% of the HR-low cases. The distribution of germline *BRCA2* mutations was more homogeneous between the three groups (1.1 % of HR + cases, 0.9% of TN cases and 1.3% of HR-low cases).

Chemotherapy was the first line treatment for 6755 patients with HR + tumors (40.3%), 1967 with TN tumors (93.1%) and 203 with HR-low tumors (89%). Chemotherapy was taxane-based in 66.7% of cases, anthracyclines-based in 32.9% of cases, and capecitabine in 18% of cases. Distribution of first line systemic treatment is described in Table S3 in supplementary data. Characteristics and outcomes of patients with HR-low tumors who received first-line ET are described in Supplementary Table S4.

### 3.2 Overall survival

**Fig. 1:**
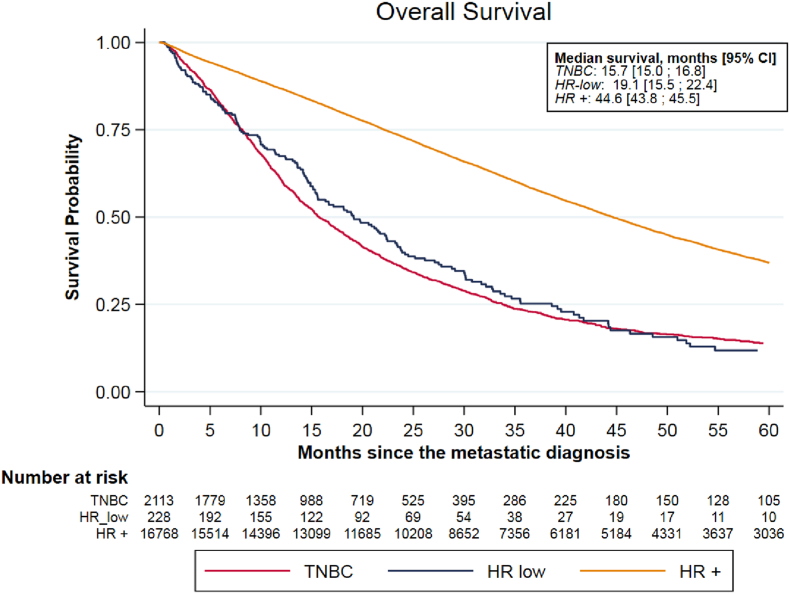
Overall survival by Hormone Receptors group.

Median OS were 44.6 months (43.8-45.5), 15.7 months (15.0-16.8) and 19.1 months (15.5-22.4) and in the HR+, TN and HR-low groups, respectively (Fig. 1). The multivariable analysis adjusted on age at MBC diagnosis, histological grade and subtype, metastatic-free interval, number and sites of metastases and HER2 status (0 *vs*. 1-2+), identified no significant OS difference between HR-low and TN groups (Hazard Ratio 0.93, 95%CI 0.77-1.11), while HR + patients had a better OS than TN patients (reference) (Hazard Ratio 0.51, 95%CI 0.48-0.54) (Table 2).

**Table 2:** **Multivariable model for Overall Survival** among patients with HER2 negative MBC for whom HR group could be calculated and with no missing value on the variable included in the model (N = 17,712).

	Multivariable associations		
	Hazard Ratio	95% CI	P-value
**HR group**			<0.001
TN	1.00	Ref.	
HR low	0.93	[0.77-1.11]	
HR +	**0.51**	**[0.48; 0.54]**	
**Age at metastatic diagnosis (categorical)**			<0.001
<50	1.00	Ref.	
[50 - 70]	**1.31**	**[1.25; 1.38]**	
>70	**1.88**	**[1.78; 1.99]**	
**Tumor Grade III at primary tumor diagnosis**			<0.001
No	1.00	Ref.	
Yes	**1.33**	**[1.27; 1.38]**	
**Histological type at primary tumor diagnosis**			<0.001
Ductal	1.00	Ref.	
Lobular	**1.26**	**[1.20; 1.33]**	
Both	1.10	[0.91; 1.32]	
Other	**0.89**	**[0.84; 0.96]**	
**Number of metastatic sites at metastasis diagnosis**			<0.001
<2	1.00	Ref.	
≥2	**1.53**	**[1.46; 1.60]**	
**Type of metastases**			<0.001
Bone or nodes metastases	1.00	Ref.	
Brain metastases	**2.08**	**[1.91; 2.26]**	
Visceral metastases	**1.25**	**[1.19; 1.30]**	
**HER2 IHC status**			<0.001
HER2 0	1.00	Ref.	
HER2 1-2+	**0.92**	**[0.88; 0.95]**	
**Metastatic-Free Interval**			<0.001
≤6 (De novo)	1.00	Ref.	
]6; 24]	**2.37**	**[2.22; 2.52]**	
]24; 72]	**1.67**	**[1.59; 1.75]**	
>72	**0.96**	**[0.92; 1.01]**	
Year of metastasis diagnosis			0.005
[2007-2009]	1.00	Ref.	
[2010-2012]	1.01	[0.95; 1.06]	
[2013-2015]	0.94	[0.89; 0.99	
[2016-2018]	1.02	[0.96; 1.09]	
[2019-2021]	1.06	[0.97; 1.16]	

Results were similar in the exploratory analysis of ER-low group. Median OS were 44.7 months (43.9-45.7) 15.7 months (15.0-16.8) and 19.5 months (15.6-24.2) in the ER+, TN and ER-low groups, respectively (Fig. S3 in supplementary data). The multivariable analysis adjusted on age at MBC diagnosis, pathological grade and subtype, metastatic-free interval, number and sites of metastases and HER2 status (0 vs. 1-2+), identified no significant OS difference between ER-low and TN groups (Hazard Ratio 0.88, 95%CI 0.76-1.02), while ER + patients had a better OS than TN patients (reference) (Hazard Ratio 0.51, 95%CI 0.48-0.54) (Table S5 in supplementary data).

### 3.3 Progression-free survival

**Fig. 2:**
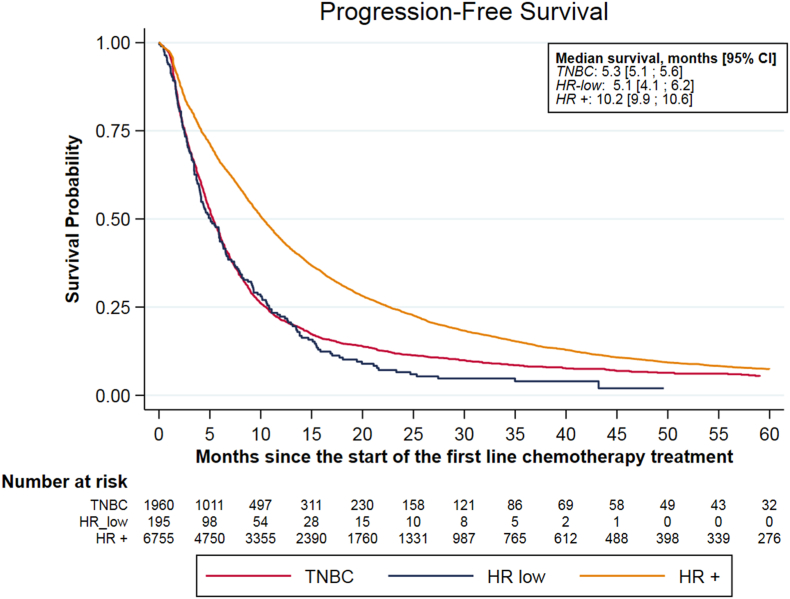
Progression free survival under first line chemotherapy, by Hormone Receptors group.

Progression free survival at the first line treatment was analyzed on the population of patients who received a first treatment line of chemotherapy (n = 8910). Median PFS under first line chemotherapy were 10.2 months (9.9-10.6), 5.3 months (5.1-5.6) and 5.1 months (4.1-6.2) for the HR+, TN and HR-low groups, respectively (Fig. 2). In the multivariable analysis adjusted on the same variables, the HR-low group was affected by a statistically lower PFS compared to TN (Hazard Ratio 1.25, 95% CI 1.04-1.49). On the other hand, the HR + group displayed a longer PFS than the TN group (Hazard Ratio 0.74, 95%CI 0.70; 0.79) (Table 3).

**Table 3:** **Multivariable model for Progression Free Survival under first line chemotherapy**, among patients with HER2 negative MBC for whom HR group could be calculated and with no missing value on the variable included in the model (N = 8318).

	Multivariable associations		
	Hazard Ratio	95% CI	P-value
**Hormone Receptors group**			<0.001
TN	1.00	Ref.	
HR low	**1.25**	**[1.04; 1.49]**	
HR +	**0.74**	**[0.70; 0.79]**	
**Age at metastatic diagnosis (categorical)**			<0.001
<50	1.00	Ref.	
[50 - 70]	**1.15**	**[1.09; 1.21]**	
>70	**1.32**	**[1.23; 1.42]**	
**Histological Grade III at primary tumor diagnosis**			<0.001
No	1.00	Ref.	
Yes	**1.14**	**[1.08; 1.20]**	
**Histological type at primary tumor diagnosis**			0.338
Ductal	1.00	Ref.	
Lobular	1.04	[0.97; 1.12]	
Both	0.90	[0.71; 1.14]	
Other	**0.98**	**[0.90; 1.07]**	
**Number of metastatic sites at start of line of treatment**			<0.001
<2	1.00	Ref.	
≥2	**1.44**	**[1.36; 1.52]**	
**Type of metastases**			<0.001
Bone or nodes metastases	1.00	Ref.	
Brain metastases	**1.66**	**[1.49; 1.84]**	
Visceral metastases	**1.21**	**[1.14; 1.28]**	
**HER2 IHC status**			<0.001
HER2 0	1.00	Ref.	
HER2 1-2+	**0.89**	**[0.85; 0.94]**	
**Metastatic-Free Interval**			<0.001
≤6 (De novo)	1.00	Ref.	
]6; 24]	**2.33**	**[2.16; 2.52]**	
]24; 72]	**1.73**	**[1.62; 1.84]**	
>72	**1.18**	**[1.11; 1.26]**	
Year of metastasis diagnosis			0.044
[2007-2009]	1.00	Ref.	
[2010-2012]	1.06	[0.99; 1.14]	
[2013-2015]	0.97	[0.90; 1.04]	
[2016-2018]	1.02	[0.95; 1.10]	
[2019-2021]	1.03	[0.93; 1.15]	

Results were similar in the exploratory analysis of ER-low group. 8854 patients received chemotherapy as first line treatment. Median PFS were 10.3 months (9.9-10.6), 5.3 months (5.1-5.6) and 5.2 months (4.0-6.3) for the ER+, TN and ER-low groups, respectively (Fig. S4 in supplementary data). In the multivariable analysis, there was no significant PFS difference between ER-low and TN groups (Hazard Ratio 1.14, 95% CI 0.98-1.32) but a longer PFS in ER + group compared to TN (Hazard Ratio 0.74, 95%CI 0.70-0.79) (Table S6 in supplementary data).

## 4 Discussion

We present here, to our knowledge, the first comprehensive analysis of HR-low tumors in a large MBC population of more than 30,000 cases. Data about the HR-low breast cancer are scarce in the metastatic stage, likely because it represents a small proportion of MBC. Our large MBC database enables us to gain a better understanding of this group, compared to TN and HR + tumors.

We found that the prognosis of these patients was similar to that of patients with TN MBC, both in terms of OS and PFS under a first line chemotherapy. The frequency of 1.2% HR-low MBC found in our cohort was similar to that of other studies [8]. Clinico-pathological characteristics were more similar to TN group than to HR + group even if the HR-low group was more heterogeneous. These data are consistent with the growing number of data in the early stage setting, showing similar profile in terms of prognosis (pCR and OS) and histopathological characteristics. As in our results, these study consistently reported more lobular histology and less grade III in the HR-low group than in the TN group [8,9,13,17,18]. In addition, distribution of metastatic site appears similar between HR-low group and TN group with more brain metastases and less bone-only disease than in HR + group. This was consistent with our previous report in an earlier version of the ESME cohort that showed that HR negative status was predictive of brain metastasis development [19]. These results support comparable behavior of HR-low group.

We found a higher frequency of *BRCA1* mutations in the HR-low group, consistent with other studies [20], even though most patients had not been tested at the time of metastatic diagnosis in our series. Indeed, germinal *BRCA1/2* testing should now be offered to all HER2 negative patients at the metastatic stage in view of treatment possibilities [21,22]. However, our large cohort covers a period of time (2008 – 2021) during which the therapeutic impact of *BRCA1/2* mutation research remains minor, since authorization for use of PARP inhibitors in this indication was obtained in 2019 in France. The percentage of germline *BRCA1/2* mutation testing has increased rapidly in recent years, and this data will need to be reassessed later.

Another issue in this population is sensitivity to ET. In France, the majority of HR-low cancers are treated as TN, with in our cohort only 29 patients having received ET in first line, which does not allow us to answer this question. In the adjuvant setting, most of the studies found no benefit of ET in this patients population [6,17,18]. It is important to note that one recent study, reporting a benefit of adjuvant hormonotherapy in a heterogeneous (stages I-IV) cohort of 431 patients, classified an unusually high percentage (17.5%) of tumors as being HR-low [7]. This high percentage of HR-low tumors, associated with the heterogeneity of the population and an impressive 0.10 Hazard Ratio reported for survival of HR-low patients receiving ET compared to not, warrant additional validation in independent datasets, considering the classical magnitude of benefit for ET, even in the HR-positive population. Another large study of more than 10,000 patients with ER-low EBC showed a decrease in OS without adjuvant ET but in a population with other factors associated with a poorer prognosis (negative PR status, higher grade, higher Ki67, neoadjuvant CT) [23].

The HR-low MBC is probably not entirely comparable from the HR-low EBC. Indeed, 66.7% of our patients presented a metachronous disease and some of them had already been treated with ET at the adjuvant stage, with possible selection of a more resistant population. In a significant number of cases, the HR status between the primary and the metastases has changed. This change has already been reported in the ESME database, where it was found in 14.2% of cases, hence the importance of reappraisal of receptors status by biopsies at the metastatic stage [24]. This evolution appeared to be more frequent in HR-low MBC, of whom a significant number were ER + at the early stage. This element may lead us to suspect a population that is more heterogeneous than at the early stage, with a proportion of patients with a possible selection linked to exposure to adjuvant chemotherapy and ET [[25], [26], [27]].

The principal strengths of the study are robust methodology of data collection and management of a nationwide comprehensive database dedicated to MBC, the large number of patients and significant amount of information collected, allowing the comprehensive analysis of a rare group of tumors in a little-studied population at the metastatic stage.

One of the limitations of our study is the small number of patients who received CDK4-6 inhibitor in this setting, partly due to the approval date of these drugs, partly due to the common consideration of this group of tumors as non-endocrine responsive. An update of the data would be interesting with more hindsight on this question, however with the increasing risk of reduction in endocrine therapies prescription in the HR-low group, considering the growing level of evidences of a TN-like clinical behavior.

Similarly, no patients were treated with immunotherapy in this timeframe. There is some evidence in the literature for the efficacy of immunotherapy in HR-low BC, including an immune landscape similar to TN. In a study of the immune landscape according to ER expression level, Voorwerk and colleagues compared the levels of stromal TILs, CD8 + T cells, PD-L1 positivity as well as expression of immune-related gene signatures [28]. Results in the groups with tumors expressing ER from 1% to 50% were similar to those of TN compared with the groups of tumors with ER above 50%. Clinically, these tumors appear to be more responsive to neoadjuvant immunotherapy at the early stage than HR + BC [16]. Recent studies showed that immunotherapy used at early stage for ER-low breast cancer was associated with pCR rates similar to TNBC [[29], [30], [31]]. This modification of treatment at the early stage could lead to a change of the metastatic population in the contemporary clinical practice and might change its prognosis compared to our analysis.

Other potential biases are classically related to its retrospective and observational design with risk of selection bias, information of misclassification bias.

Considering this growing corpus of evidence, in the EBC as well as in the MBC setting, of clinical characteristics and prognosis of HR-low tumors closely related to TN tumors, inclusion of this group of patients in clinical trials and clinical guidelines dedicated to TN patients appears logical and would offer a growing number of therapeutic options in a population affected with low, if any, endocrine sensitivity.

## 5 Conclusion

In this large cohort, patients with HER2-non overexpressed, HR-low MBC seem to share clinicopathological characteristics and prognostic features with patients bearing triple negative breast cancer, and should probably be considered as so in term of risk stratification and therapeutic options.

## 6 Final considerations

### Sponsors

The ESME MBC database receives financial support from industrial partners. Unicancer is the sole data controller for data processing and manages the ESME MBC database (data collection, analysis and publication) independently.

## CRediT authorship contribution statement

**Marie Alexandre:** Writing – original draft, Investigation, Conceptualization. **Eva Teruel:** Methodology, Formal analysis. **Florence Dalenc:** Writing – review & editing, Investigation. **Thibaut de la Motte Rouge:** Writing – review & editing, Investigation. **Etienne Brain:** Writing – review & editing, Investigation. **Vincent Massard:** Writing – review & editing, Investigation. **Monica Arnedos:** Writing – review & editing, Investigation. **Audrey Mailliez:** Writing – review & editing, Investigation. **Jean-Marc Ferrero:** Writing – review & editing, Investigation. **Thomas Bachelot:** Writing – review & editing, Investigation. **Isabelle Desmoulins:** Writing – review & editing, Investigation. **Marie-Ange Mouret-Reynier:** Writing – review & editing, Investigation. **Christelle Levy:** Writing – review & editing, Investigation. **Antony Gonçalves:** Writing – review & editing, Investigation. **Anca Berghian:** Writing – review & editing, Investigation. **Thierry Petit:** Writing – review & editing, Investigation. **Jean-Sébastien Frenel:** Writing – review & editing, Investigation. **Anne-Laure Martin:** Writing – review & editing, Supervision. **Thomas Grinda:** Writing – review & editing, Investigation. **William Jacot:** Writing – review & editing, Methodology, Investigation, Conceptualization.

## Declaration of competing interest

Marie Alexandre reports support for attending meetings or travel from Novartis, Gilead, Lilly, MSD, Pfizer, Participation on a Data Safety Monitoring Board or Advisory Board from Novartis, AstraZeneca, payment or honoraria for presentations from Gilead, Lilly.

Eva Teruel reports no conflict of interest.

Florence Dalenc reports support for attending meetings or travel from Novartis, Roche, Gilead, Participation on a Data Safety Monitoring Board or Advisory Board from Novartis, Roche, Mennarini.

Thibaut de la Motte Rouge reports grants from Seagen, consulting fees from Abbvie, AstraZeneca, GSK, Roche, Sanofi, Pfizer, Regeneron, Novartis, Gilead, Mennarini, Eisai, support for attending meetings or travel from MSD, Gilead, Pfizer, Esai, other services from Natera.

Etienne Brain reports consulting fees from Exact Sciences, Mennarini, Pfizer, payment or honoraria for presentations from AstraZeneca, Daiichi, Eli Lilly, Exact Sciences, Incyte, Novartis, Pfizer, Takeda, support for attending meetings or travel from AstraZeneca, Daiichi, Exact Sciences, Gilead, Novartis, Pfizer.

Vincent Massard reports Mennarini, AstraZeneca, Lilly, support for attending meetings or travel from AstraZeneca, Lilly, Novartis, participation on an advisory board from AstraZeneca, Lilly, Novartis.

Monica Arnedos reports consulting fees from Novartis, Gilead, AstraZeneca, Daiichi-Sankyo, Eli-Lilly, Mennarini, Pfizer, payment or honoraria for presentations from Novartis, Pfizer, AstraZeneca, support for attending meetings or travel from Novartis, Eli-Lilly, AstraZeneca.

Audrey Mailliez reports no conflict of interest.

Jean-Marc Ferrero reports no conflict of interest.

Thomas Bachelot reports grants from AstraZeneca, Pfizer, SeaGen, Novartis, payment or honoraria for presentations from Novartis, Daiichi Sankyo, Pfizer, Lilly, support for attending meetings or travel from Roche, Daiichi Sankyo, AstraZeneca, Pfizer, Novartis, participation on a Data Safety Monitoring Board or Advisory Board from AstraZeneca, SeaGen, Novartis, Pfizer, Lilly, Daiichi Sankyo.

Isabelle Desmoulins reports no confict of interest.

Marie-Ange Mouret-Reynier reports no conflict of interest.

Christelle Levy reports support for attending meetings or travel from Novartis.

Anthony Gonçalves reports consulting fees from Novartis, Gilead, Daiichi Sankyo, MSD, Astra Zeneca, support for attending meetings or travel from Mylan.

Anca Berghian reports no confict of interest.

Thierry Petit reports consulting fees from Pfizer, Novartis, Lilly, Daiichi Sankyo, support for attending meetings or travel from Pfizer, Novartis, Gilead, participation on a Data Safety Monitoring Board or Advisory Board from Pfizer, Mennarini.

Jean-Sébastien Frenel reports consulting fees from AstraZeneca, GSK, Esai, MSD, Lilly, Pfizer, Novartis, Daichi Sankyon, Seagen, ABBVIE, ROCHE, Pharma End, Boehringer, payment or honoraria for presentations from Astra Zeneca, GSK, Esai, MSD, Lilly, Pfizer, Novartis, Daichi Sankyon, Seagen, Abbvie, Astra Zeneca, GSK, Esai, MSD, Lilly, Pfizer, Novartis, Daichi Sankyon, Seagen, Abbvie, participation on a Data Safety Monitoring Board or Advisory Board from Astra Zeneca, GSK, Esai, MSD, Lilly, Pfizer, Novartis, Daichi Sankyon, Seagen, Abbvie.

Anne-Laure Martin reports no conflict of interest.

Thomas Grinda reports grants from foundation Philippe, consulting fees from Pfizer, Lilly, Astrazeneca, Gilead, payment or honoraria for presentations from Lilly and Roche, support for attending meetings or travel from Gilead, Astrazeneca, Pfizer and Lilly.

William Jacot reports grants, personal fees, and non-financial support from AstraZeneca, grants and personal fees from Daiichi Sankyo, personal fees and non-financial support from Eisai, personal fees and non-financial support from Novartis, personal fees and non-financial support from Roche, personal fees and non-financial support from Pfizer, personal fees and non-financial support from Eli Lilly, personal fees from MSD, personal fees from BMS, personal fees and non-financial support from Chugai, personal fees from Seagen, and personal fees and non-financial support from Gilead outside the submitted work.
