# Supplementary material for: Characteristics and outcomes of patients with HER2-negative metastatic breast cancers with low expression of estrogen and progesterone receptors in the multicenter ESME cohort
Source: Breast. 2026 May 18;88:104815. doi: 10.1016/j.breast.2026.104815 (PMC13213639; doi:10.1016/j.breast.2026.104815)
Supplement: Multimedia component 1 [file mmc1.docx]

**Supplementary data**

**Supplementary Figure S1. Flowchart of patient selection.**

**Exclusion regarding HER2 status (N =6,905)**

Undetermined HER2 status (N= 1,744)

HER2 positive patient (N= 5,161)

Population ESME MBC

(N = 30,459)

HER2 negative patients

(N=23,554)

HER2 negative patients with available ER percentage (N=19,226)

**Exclusion regarding HR status (N = 4,328)**

Undetermined HR status (N= 80)

Undetermined ER percentage (N= 4,248)

- ER percentage missing (N=3,868)
- Bilateral tumor with duplicates last histology on primitive tumor (N=354)
- Duplicates last histology on primitive tumor with conflicting result (N=26)

HER2 negative patients with HR group available (N=19,109)

**Primary objective analysis population**

Patients that could not be classified due to missing PR percentage (N = 117)

**Supplementary Table S1: Distribution of systemic treatment at early stage for non de novo metastatic breast cancer.**

|  | **TNBC  N = 2,113** | **HR Low  N = 228** | **HR+  N = 16,768** | **All  N = 19,109** |
| --- | --- | --- | --- | --- |
| Non de novo metastatic breast cancer, n (%) | 1,470 (69.6%) | 156 (68.4%) | 11,126 (66.4%) | 12,752 (66.7%) |
| Chemotherapy alone | 1,099 (81.3%) | 88 (60.7%) | 750 (7.3%) | 1,937 (16.4%) |
| Combination of chemotherapy and endocrine therapy | 194 (14.4%) | 41 (28.3%) | 6,765 (65.8%) | 7,000 (59.4%) |
| Endocrine therapy alone | 58 (4.3%) | 16 (11.0%) | 2,769 (26.9%) | 2,843 (24.1%) |
| Missing | 119 | 11 | 842 | 972 |

**Supplementary Table S2: Latest available biopsy (phenotype source) and comparison with the primary tumor status in case of metastasis-based phenotype and availability of the primary tumor-based phenotype.**

|  | **TNBC  N = 2,113** | **HR Low  N = 228** | **HR+  N = 16,768** | **All  N = 19,109** |
| --- | --- | --- | --- | --- |
| Phenotype source, n (%) |  |  |  |  |
| Metastasis-based phenotype | 758 (35.9%) | 109 (47.8%) | 7,790 (46.5%) | 8,657 (45.3%) |
| Primary tumor-based phenotype | 1,355 (64.1%) | 119 (52.2%) | 8,978 (53.5%) | 10,452 (54.7%) |
| If metastasis-based phenotype, primary tumor-based phenotype available ? , n (%) | 558 (73.6%) | 84 (77.1%) | 5,992 (76.9%) | 6,634 (76.6%) |
| Primary tumor-based phenotype, n (%) |  |  |  |  |
| HR Low | 22 (3.9%) | 18 (21.4%) | 15 (0.3%) | 55 (0.8%) |
| HR+ | 182 (32.6%) | 50 (59.5%) | 5,925 (98.9%) | 6,157 (92.8%) |
| TNBC | 354 (63.4%) | 16 (19.0%) | 52 (0.9%) | 422 (6.4%) |

Supplementary Table S3: Distribution of first line treatment.

|  | **HR group** | | | | | |
| --- | --- | --- | --- | --- | --- | --- |
|  | **TN** | | **HR low** | | **HR +** | |
|  | **N=2,113** | | **N=228** | | **N=16,768** | |
| **Type of treatment as first line** |  |  |  |  |  |  |
| Chemotherapy | 1,967 | (93.1%) | 203 | (89.0%) | 6,755 | (40.3%) |
| Endocrine therapy | 81 | (3.8%) | 21 | (9.2%) | 9,894 | (59.0%) |
| No first line treatment | 65 | (3.1%) | 4 | (1.8%) | 119 | (0.7%) |
| **First line chemotherapy treatment** |  |  |  |  |  |  |
| **Taxanes** | 1,182 | (60.3%) | 116 | (59.5%) | 4,649 | (68.8%) |
| **Anthracyclines** | 459 | (23.4%) | 49 | (25.1%) | 2,420 | (35.8%) |
| **Capecitabine** | 474 | (24.2%) | 39 | (20.0%) | 1,090 | (16.1%) |
| **Vinorelbine** | 63 | (3.2%) | 5 | (2.6%) | 171 | (2.5%) |
| **Gemcitabine** | 121 | (6.2%) | 14 | (7.2%) | 76 | (1.1%) |
| **Eribulin** | 53 | (2.7%) | 4 | (2.1%) | 42 | (0.6%) |
| **Cyclophosphamide** | 528 | (26.9%) | 63 | (32.3%) | 2,399 | (35.5%) |
| **Etoposide** | 9 | (0.5%) | 1 | (0.5%) | 28 | (0.4%) |
| **Platinium Derivatives** | 321 | (16.4%) | 31 | (15.9%) | 187 | (2.8%) |
| **First line endocrine therapy treatment** |  |  |  |  |  |  |
| **Aromatase Inhibitors** | 60 | (68.2%) | 23 | (79.3%) | 7,870 | (79.5%) |
| **Fulvestrant** | 8 | (9.1%) | 3 | (10.3%) | 1,513 | (15.3%) |
| **Megestrol** | 0 | (0.0%) | 0 | (0.0%) | 8 | (0.1%) |
| **Associated with targeted therapy** | 8 | (9.1%) | 4 | (13.8%) | 2,470 | (25.0%) |
| **Palbociclib** | 7 | (87.5%) | 4 | (100.0%) | 1,821 | (73.7%) |
| **Abemaciclib** | 1 | (12.5%) | 0 | (0.0%) | 154 | (6.2%) |
| **Ribociclib** | 0 | (0.0%) | 0 | (0.0%) | 269 | (10.9%) |
| **Everolimus** | 0 | (0.0%) | 0 | (0.0%) | 283 | (11.5%) |
| **Alpelisib** | 0 | (0.0%) | 0 | (0.0%) | 22 | (0.9%) |

**Supplementary Table S4: Characteristics and outcomes of patients with HR-low tumors who received first-line endocrine therapy.**

|  | **N = 21** |
| --- | --- |
| Age at metastatic diagnosis |  |
| Mean (SD) | 67.9 (12.6) |
| Min - Max | 43.8 - 90.3 |
| Missing | 1 |
| Type of metastases, n (%) |  |
| Non visceral metastasis | 13 (61.9%) |
| Brain visceral metastasis | 2 (9.5%) |
| Non brain visceral metastasis | 6 (28.6%) |
| Median OS, months (95% CI) | 16.7 (13.3-52.3) |
| Median PFS, months (95% CI) | 4.1 (2.0-6.3) |

**Supplementary Figure S2. Flow chart for the ER-low exploratory analysis population.**

**Exclusion regarding HER2 status (N =6,905)**

- Undetermined HER2 status (N= 1,744)
- HER2 positive patient (N= 5,161)

ESME MBC population

(N = 30,459)

HER2 negative patients

(N=23,554)

HER2 negative patients with available ER percentage (N=19,226)

**Exclusion regarding HR status (N = 4,328)**

- Undetermined HR status (N= 80)
- Unavailable ER percentage (N= 4,248)
  - ER percentage missing (N=3,868)
  - Bilateral tumor with duplicates last histology on primitive tumor (N=354)
  - Duplicates last histology on primitive tumor with conflicting result (N=26)

HER2 negative patients with ER group available (N=19,046)

**Exploratory analysis population**

Patients ER 0 with PR percentage > 0 or ER 0 with PR percentage missing (N = 180)

**Supplementary Figure S3.** **Overall survival by ER group.**


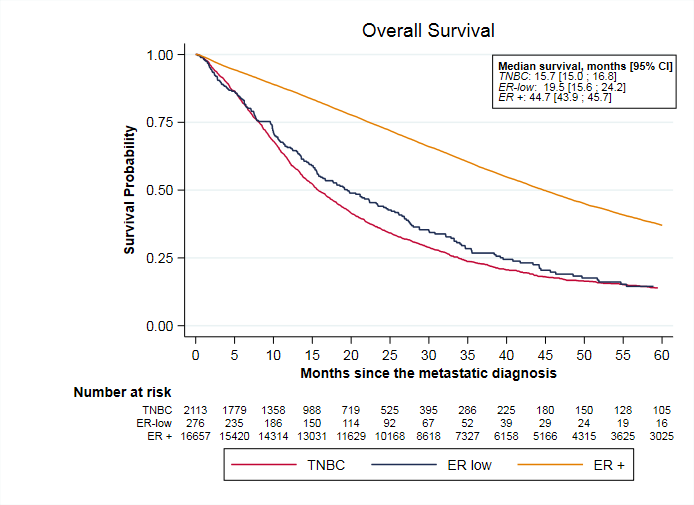


**Supplementary Table S5. Multivariable model for Overall Survival.** Selected HER2 negative patients for whom ER group could be calculated and with no missing value on the variable included in the model (N=17,848).

|  | **Multivariable associations** | | |
| --- | --- | --- | --- |
|  | **HR** | **95% CI** | **P-value** |
| **ER group** |  |  | <0.001 |
| TNBC | 1.00 | Ref. |  |
| ER low | 0.90 | [0.77 ; 1.04] |  |
| ER + | **0.50** | **[0.47 ; 0.53]** |  |
| **Age at metastatic diagnosis (categorical)** |  |  | <0.001 |
| < 50 | 1.00 | Ref. |  |
| [50 - 70] | **1.29** | **[1.23 ; 1.36]** |  |
| > 70 | **1.75** | **[1.65 ; 1.85]** |  |
| **Histological Grade III at primary tumor diagnosis** |  |  | <0.001 |
| No | 1.00 | Ref. |  |
| Yes | **1.33** | **[1.27 ; 1.38]** |  |
| **Histological type at primary tumor diagnosis** |  |  | <0.001 |
| Ductal | 1.00 | Ref. |  |
| Lobular | **1.28** | **[1.21 ; 1.35]** |  |
| Both | 1.08 | [0.89 ; 1.31] |  |
| Other | **0.91** | **[0.85 ; 0.98]** |  |
| **Number of metastatic site at metastasis diagnosis** |  |  | <0.001 |
| <2 | 1.00 | Ref. |  |
| >=2 | **1.54** | **[1.47 ; 1.61]** |  |
| **Type of metastasis** |  |  | <0.001 |
| Non visceral metastasis | 1.00 | Ref. |  |
| Brain visceral metastasis | **2.09** | **[1.92 ; 2.28]** |  |
| Non brain visceral metastasis | **1.26** | **[1.21 ; 1.32]** |  |
| **HER2 IHC status** |  |  | <0.001 |
| HER2 0 | 1.00 | Ref. |  |
| HER2 1-2+ | **0.91** | **[0.87 ; 0.94]** |  |
| **Metastatic-Free Interval (months)** |  |  | <0.001 |
| ≤ 6 (De novo) | 1.00 | Ref. |  |
| ]6 ; 24] | **2.40** | **[2.24 ; 2.56]** |  |
| ]24 ; 72] | **1.67** | **[1.59 ; 1.76]** |  |
| > 72 | **0.95** | **[0.90 ; 1.00]** |  |

**Supplementary Figure S4. Progression-Free Survival by ER group.**


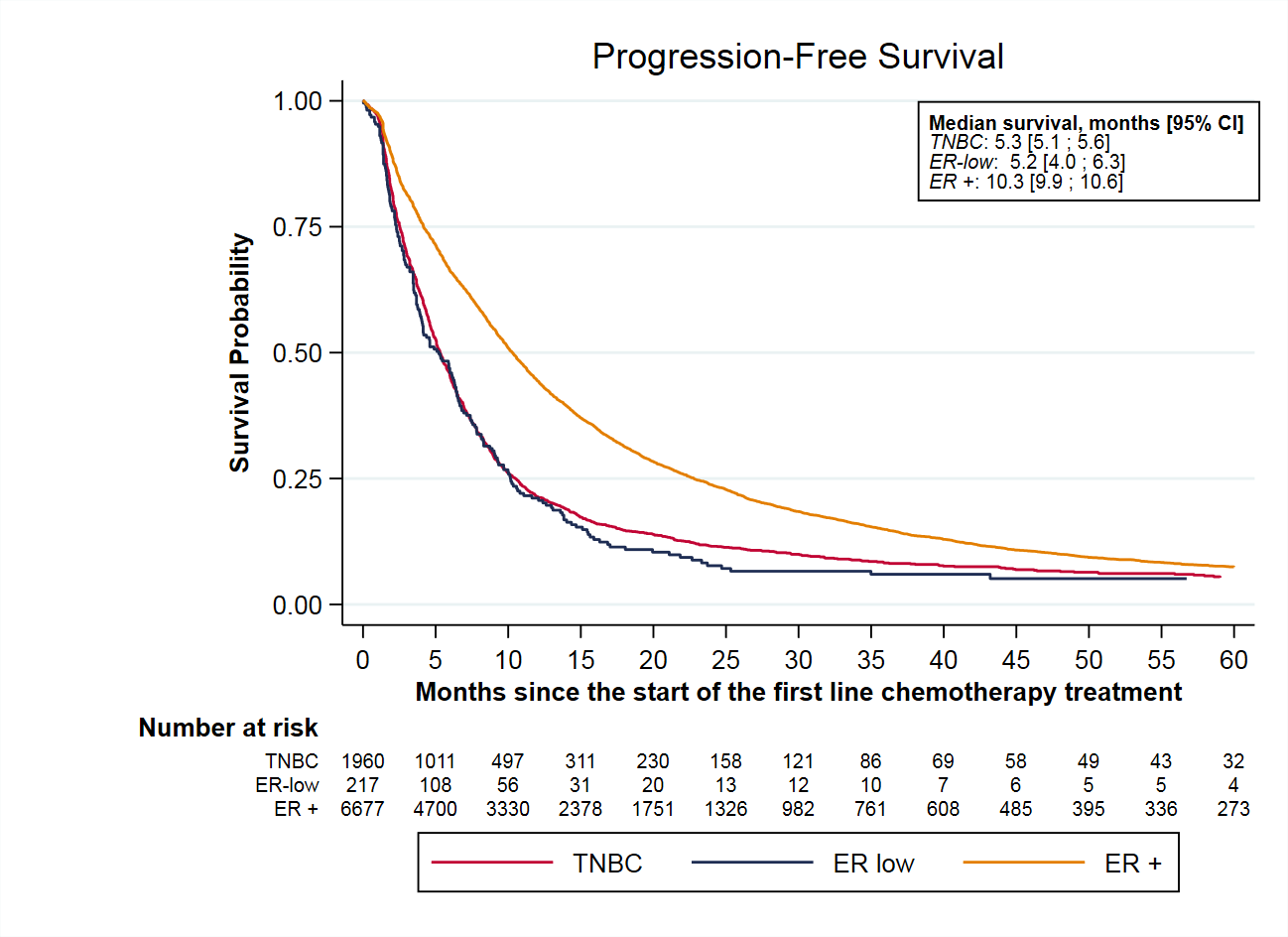


**Supplementary Table S6. Multivariable model for Progression-Free Survival. Selected HER2 negative patients for whom ER group could be calculated and with no missing value on the variable included in the model (N=8318).**

|  | **Multivariable associations** | | |
| --- | --- | --- | --- |
|  | **HR** | **95% CI** | **P-value** |
| **ER group** |  |  | <0.001 |
| TNBC | 1.00 | Ref. |  |
| ER low | **1.23** | **[1.04 ; 1.46]** |  |
| ER + | **0.74** | **[0.70 ; 0.79]** |  |
| **Age at metastatic diagnosis (categorical)** |  |  | <0.001 |
| < 50 | 1.00 | Ref. |  |
| [50 - 70] | **1.14** | **[1.08 ; 1.20]** |  |
| > 70 | **1.29** | **[1.20 ; 1.39]** |  |
| **Histological Grade III at primary tumor diagnosis** |  |  | <0.001 |
| No | 1.00 | Ref. |  |
| Yes | **1.14** | **[1.08 ; 1.20]** |  |
| **Histological type at primary tumor diagnosis** |  |  | 0.339 |
| Ductal | 1.00 | Ref. |  |
| Lobular | 1.04 | [0.96 ; 1.12] |  |
| Both | 0.85 | [0.67 ; 1.09] |  |
| Other | 0.97 | [0.89 ; 1.06] |  |
| **Number of metastatic site at start of line of treatment** |  |  | <0.001 |
| <2 | 1.00 | Ref. |  |
| >=2 | **1.48** | **[1.41 ; 1.57]** |  |
| **Type of metastasis** |  |  | <0.001 |
| Non visceral metastasis | 1.00 | Ref. |  |
| Brain visceral metastasis | **1.62** | **[1.46 ; 1.80]** |  |
| Non brain visceral metastasis | **1.20** | **[1.13 ; 1.27]** |  |
| **HER2 IHC status** |  |  | <0.001 |
| HER2 0 | 1.00 | Ref. |  |
| HER2 1-2+ | **0.89** | **[0.85 ; 0.94]** |  |
| **Metastatic-Free Interval** |  |  | <0.001 |
| ≤ 6 (De novo) | 1.00 | Ref. |  |
| ]6 ; 24] | **2.31** | **[2.14 ; 2.49]** |  |
| ]24 ; 72] | **1.71** | **[1.61 ; 1.82]** |  |
| > 72 | **1.15** | **[1.08 ; 1.23]** |  |
